# Agreement between 3D Motion Analysis and Tele-Assessment Using a Video Conferencing Application for Telerehabilitation

**DOI:** 10.3390/healthcare9111591

**Published:** 2021-11-20

**Authors:** Kyeongjin Lee

**Affiliations:** Department of Physical Therapy, College of Health Science, Kyungdong University, Wonju 24764, Korea; kjlee@kduniv.ac.kr

**Keywords:** telerehabilitation, mobile application, kinematics, validity, reliability

## Abstract

The global pandemic of the coronavirus disease 2019 (COVID-19) has highlighted the need for remote healthcare services. This study aimed to evaluate the concurrent validity and reliability of tele-assessment using 3D motion analysis and video conferencing applications. The subjects of this study were 14 Pilates instructors and 14 healthy adults, who repeated five exercises of “side spine stretch”, “bridge”, “toe taps”, “quadruped leg raise”, and “cat and cow” five times each. We performed 3D kinematic analysis with 16 infrared cameras while the subject performed each exercise, and the image captured by one webcam was transmitted to the evaluators through a video conferencing application, and eight raters evaluated the mobility, stability, and symmetry of the movement. The result was then compared with the gold standard 3D motion analysis to evaluate the teleassessment system. The concurrent validity of the data obtained using both methods was analyzed. In addition, the inter-rater reliability of the data from the eight raters was evaluated. As a result, mobility showed excellent (ICC > 0.75, ICCs: intraclass correlation coefficients) or good agreement (ICC = 0.6–0.74) with 3D motion analysis and tele-assessment in all motions. The analysis of stability showed high agreement in general, but it was not significant in “cat and cow.” Symmetry showed moderate agreement only in “bridge” and “toe taps”, showing low agreement compared to other components. In addition, the inter-rater reliability of the tele-assessment showed good agreement (ICC = 0.744). Although there were few components with weaker agreements, the results of this study confirmed that it is a valid and reliable method of tele-assessment using video conferencing applications and showed feasibility as an alternative to the existing face-to-face examination.

## 1. Introduction

The recent coronavirus (COVID-19) pandemic and its aftermath had a significant impact on the medical environment. Governments worldwide implemented restrictions such as social distancing, quarantines, and lockdowns to prevent the spread of the disease [1]. Recently, advanced countries have increased vaccinations and implemented policies to coexist with the virus, allowing people to visit hospitals without hesitation [2]. However, most of the remaining countries are still struggling with inpatient visits because of the risk of infection [1,2]. Rehabilitation and other medical services to improve general health conditions, such as musculoskeletal care, have been postponed because they are not urgent. As the accessibility to medical care decreases, patients with musculoskeletal disorders or disabilities that require close contact with health care professionals may suffer from worsening and chronicization of the disorders [2]. Lately, treatments without direct contact, such as telerehabilitation, are emerging as they can enhance the quality of treatment and life for patients with musculoskeletal disorders [3,4].

Telerehabilitation or telehealth comprises medical care and professionals that adopt information and communication technologies (ICT). Healthcare professionals can provide assessment, diagnosis, goal setting, treatment education, and monitoring through remote devices or communication skills [5]. Compared to the former in-contact treatment, telerehabilitation has immediate access and low cost, which recognizes it as a compulsory medical service for isolated regions [6]. Despite the rapid development of the technologies of telerehabilitation and remote assessment in the past decade, however, the commercialization of telerehabilitation was limited owing to social conditions and backlashes from a portion of health professionals [1,7,8]. The pandemic after COVID-19 has compelled health professionals to use telemedicine, and the World Confederation for Physical Therapy (WCPT) has also offered the use of telerehabilitation to cope with the changing medical environment [1,2]. North America, Australia, and European countries have been expanding their resources and recommendations for the use of diverse telemedical care [1]. It is almost impossible to cease telemedical care, even after the world has overcome the virus.

Video conferencing applications such as zoom, skype, and Microsoft teams have been utilized by health care professionals to meet patients online. However, treatments at home may have limited quality compared to the setting of physical therapy clinics, and treatments such as manual therapy that require close contact with patients have practical limitations [4,9]. It is nearly impossible to proceed with important special tests and palpation that are required to assess and diagnose patients without direct contact, and reliability is not provided for medical decisions made only with online interviews and examinations. However, to determine prognosis, the reliability and validity of posture or movement assessment during online interviews are essential to determine prognosis.

Despite this fact, there are only few studies to date that have validated observational motion analysis through telehealth systems compared to the results of kinematic analysis [10,11]. Therefore, this study aimed to compare posture and movement evaluation data obtained with a conferencing application to results of kinematic analysis and assess concurrent validity and inter-rater reliability to confirm the feasibility of telerehabilitation.

## 2. Materials and Methods

### 2.1. Subjects

The subjects of this study included 14 Pilates instructors and 14 healthy adults, and each group had 7 men and 7 women. They were recruited through online advertisements at an university located in Seoul. Exclusion criteria were those who had an orthopedic or neurological disease within the past year or those who had difficulty performing exercise owing to current musculoskeletal or nervous system abnormalities, pain or inflammation, and visual and hearing problems. Before starting the study, we explained the purpose and process of the study to the subjects, and after the subjects had sufficient understanding, informed consent was signed. This study was approved by the institutional review board of Kyungdong University.

### 2.2. Sample Size Calculation

The sample size calculation was based on the method described by Walter et al. [12]. Considering a significance level of 0.05, a statistical power of 0.8, a minimum acceptable reliability (intraclass correlation coefficient, ICC) (p0) of 0.5, and an expected reliability (ICC) (p1) of 0.8, 28 subjects were required. Therefore, 28 subjects were included in this study.

### 2.3. Experimental Exercise Procedures

Of the 50 volunteers, 28 who met the inclusion criteria were included. All procedures were performed in a gym located at a university in Seoul, South Korea. In this study, a total of five exercises were selected considering movements of the arms, legs, and trunk: the side spine stretch, bridge, toe taps, quadruped leg raise, and cat and cow. The subjects spent enough time watching demonstrative videos of a professional instructor and practiced as much as they needed. Each subject performed all exercises while listening to the metronome rhythms. The side spine stretch, toe taps, and quadruped leg raise were repeated 10 times: five times on the left and five times on the right, while bridge and cat and cow exercises were repeated five times.

### 2.4. Tele-Assessment Using Video Conference Application

**Figure 1:**
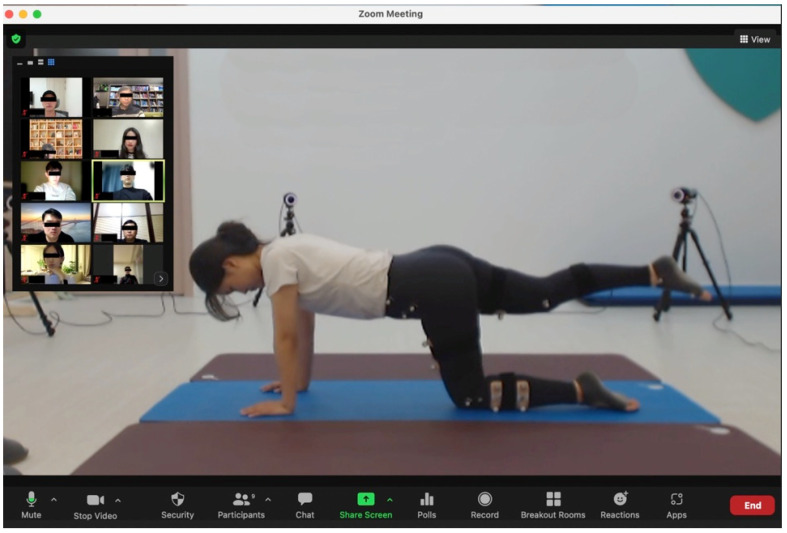
Video conference application for Tele-assessment.

To evaluate the inter-rater reliability of the tele-assessment, eight physical therapists participated as raters. Of the eight physical therapists, two specialized in musculoskeletal physical therapy with more than five years of experience, two of them had Pilates licenses and more than five years of experience, two specialized in neurological physical therapy with more than five years of experience, and the other two had less than three years of experience. The raters used a video conference application (Zoom cloud meeting, Zoom video communication, San Jose, CA, USA) for the evaluation. Each subject was streamed with a webcam (C930e, Logitech, Lausanne, Switzerland) (Figure 1).

### 2.5. Three-Dimensional Motion Analysis

The validity of the tele-assessment was evaluated using kinematic data from a three-dimensional motion capture system consisting of 16 infrared cameras (Qualisys Miqus M3, Exave AB, Göteborg, Sweden). A total of 48 reflective markers were used and placed on the following bony anatomical landmarks of the body: spinous process of the 7th cervical vertebra, acromions, medial and lateral epicondyles of elbows, styloid process of the ulna, radius, anterior superior iliac spines (ASIS), iliac crests, posterior superior iliac spines (PSIS), greater trochanters, sacrum, lateral and medial condyles of the knees, lateral malleolus of the ankles, heel, distal head of 1st metatarsal, distal head of 2nd metatarsal, distal head of 5th metatarsal, and proximal base of 5th metatarsal. Clusters consisting of four markers were used on the thigh and calf. The data obtained from the cameras were analyzed using Qualisys Track Manager software (Qualisys, Qualisys AB, Sweden).

### 2.6. Exercise Components Analysis

The subjects performed five exercises, and eight raters evaluated the mobility, stability, and symmetry components of the exercise simultaneously. These components are listed in Table 1. Each component was rated on a 10-point scale rating, with 1 point if they strongly disagreed and 10 points if they strongly agreed. Depending on the question, the points were reverse coded during analysis.

### 2.7. Statistical Analysis

All statistical measures were calculated using MedCalc^®^ statistical software (v. 20.014, MedCalc^®^ Software Ltd., Ostend, Belgium). Inter-rater reliability and concurrent validity of the tele-assessment using the video conference application were measured with intraclass correlation coefficients (ICCs) with 95% confidence intervals (CIs). In addition, a two-way mixed-effect model based on single measures and consistency was used to assess inter-rater reliability and concurrent validity (ICC3, 1). This model was selected because the selected raters were the only raters of interest. The results of this model only represent the reliability of the specific raters involved in the reliability experiment. The comparability between 3D motion analysis and tele-assessment was visualized using Bland–Altman plots and scatter plots. Statistical significance was set at *p* < 0.05.

## 3. Results

A total of 28 healthy adults (14 men and 14 women) were recruited for the study. The age of the subjects ranged from 20 to 29 years old, 23.39 ± 2.36 years. Height was 169.36 ± 8.09 cm (range, 156–182 cm), mass 62.86 ± 12.80 kg (range, 45–84 kg), and body mass index 21.69 ± 2.76 kg/m^2^ (range 15–26 kg/m^2^).

### 3.1. Concurrent Validity of the Kinematic Parameters for Subjects Using the 3D Motion Analysis System and Tele-Assessment Using a Video Conference Application

**Figure 2:**
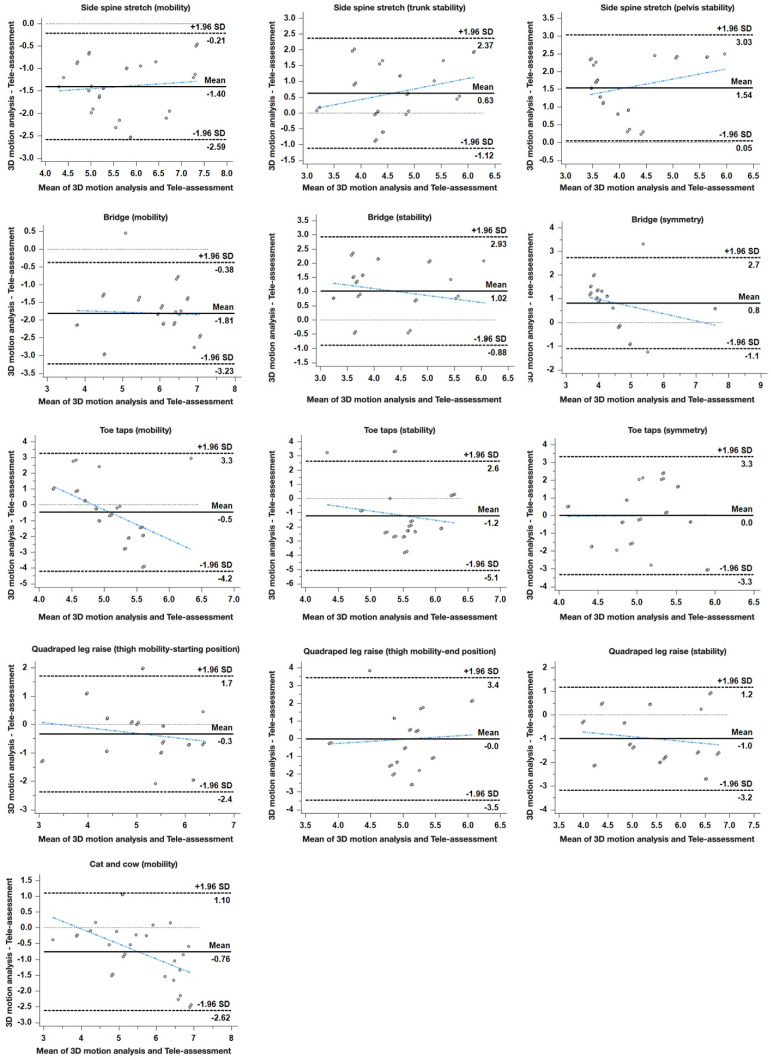
The Bland–Altman plot shows the agreement between 3D motion analysis and Tele-assessment using a video-conferencing application. The bold lines represent the mean difference between the two methods. The dashed lines represent 95% limits of agreement. Each row is an agreement according to the three elements of each exercise.

Table 2 shows the mean and standard deviation of kinematic parameters for subjects using the 3D motion analysis system and tele-assessment using the video conference application of the five exercises (Figure 2). The side spine stretch showed a high level of concurrent validity, except for trunk symmetry (trunk mobility, ICC_3.1_ = 0.884; trunk stability, ICC_3.1_ = 0.668; and pelvic stability, ICC_3.1_ = 0.763). The bridge showed a high level of concurrent validity in all components (trunk mobility, ICC_3.1_ = 0.820; leg stability, ICC_3.1_ = 0.753; and pelvic symmetry, ICC_3.1_ = 0.667). The toe taps showed a moderate level of concurrent validity in all components (mobility of the knee joint, ICC_3.1_ = 0.573; pelvic stability, ICC_3.1_ = 0.628; and leg symmetry, ICC_3.1_ = 0.564). The quadruped leg raise showed a moderate level of concurrent validity, except for the symmetry of the hip joint (thigh mobility at the starting position, ICC_3.1_ = 0.668; thigh mobility at the end position, ICC_3.1_ = 0.544; and leg stability, ICC_3.1_ = 0.611). In the cat and cow, only pelvic mobility showed a high level of concurrent validity (ICC_3.1_ = 0.811).

### 3.2. Inter-Rater Reliability of the Raters in Tele-Assessment Using Video Conference Application

Table 3 shows the mean of each component of the five exercises of the eight raters.

In the side spine stretch, only trunk mobility showed a moderate level of inter-rater reliability (ICC_3.1_ = 0.541). The bridge showed a moderate level of inter-rater reliability in all components (trunk mobility, ICC_3.1_ = 0.560; leg stability, ICC_3.1_ = 0.634; and pelvic symmetry, ICC_3.1_ = 0.551). The toe taps showed a moderate level of inter-rater reliability, except for leg symmetry (knee joint mobility, ICC_3.1_ = 0.583; pelvic stability, ICC_3.1_ = 0.711; and leg symmetry, ICC_3.1_ = 0.423). The quadruped leg raise showed a high level of inter-rater reliability in all components (thigh mobility at the starting position, ICC_3.1_ = 0.639; thigh mobility at the end position, ICC_3.1_ = 0.478; leg stability, ICC_3.1_ = 0.537; and leg symmetry, ICC_3.1_ = 0.678). The cat and cow showed a high level of inter-rater reliability, except for leg stability (pelvic mobility, ICC_3.1_ = 0.801; hip joint stability, ICC_3.1_ = 0.574; and leg stability, ICC_3.1_ = 0.242).

## 4. Discussion

We are living in a transitional period in which all lifestyles are converted to noncontact services. Tele-service has become a topic in the fields of education, financial and business transactions, and healthcare services [7,9]. The existing telehealthcare service is known only for its convenience with regards to time and place but is becoming an irreplaceable service provider after the COVID-19 pandemic [2,13]. Telehealthcare services have been classified into two categories: telecare and telehealth services [14]. Most telehealthcare has been focused on the efficacy and feasibility of a newly developed device, remote monitoring, or site-to-site telemedicine consultation [15]. Telecare has provided services including follow-up visits, management of chronic conditions, and medication management for patients with Parkinson’s disease, spinal cord injury (SCI), stroke, and Alzheimer’s disease [9,16,17,18]. Most recently, telecare consumers have expanded to healthy individuals who desire the prevention of disorders and personal healthcare. In turn, video-conferencing-apps-focused telehealth strategies monitor overall health conditions and body functions and emphasize that the preventive view of treatment goals are being noticed [9,15].

In rehabilitation, telehealth has been proven to be effective through methods using specially designed devices and networks and in virtual reality [19,20,21]. Such telerehabilitation has positively affected overall body function and mental and social health, but limitations remain with physical assessments without contacting patients [4]. Existing physical assessments require a hands-on examination from a therapist to a patient. However, since this is not possible in telerehabilitation, approaches to tele-assessment and reliability and validity for the assessment are required. This study aimed to use 3D motion analysis as a gold standard and compare it to posture and exercise evaluations proceeded through a conferencing application and to confirm the validity of the tele-assessment. In addition to that, inter-rater reliability was assessed to verify feasibility of the assessment. The results showed strong concurrent validity when compared to tele-assessment and 3D kinematic analysis.

The mobility of each joint is required to move smoothly at a constant velocity for a fine movement to occur [22]. Stability is also needed to support the trunk by limiting unnecessary movement from the joints [23]. Physical function is favorable when there is a good quality of movement with minimum postural sway and fine symmetry of the body with the distribution of weight to specific parts of the body [24]. In this study, five exercises were evaluated for mobility, symmetry, and stability factors, and the validity of tele-assessment showed different aspects depending on the factors. The analysis of mobility showed excellent (ICC > 0.75) or good agreement (ICC = 0.6 − 0.74) between 3D motion analysis and tele-assessment. The ICC for the side spine stretch was >0.884, >0.820 for bridge, >0.811 for cat and cow, >0.628 for toe taps, and >0.668 for quadruped leg raise. The analysis of mobility showed a stronger ICC compared to the analysis of stability or symmetry. There was strong evidence to support the feasibility of tele-exercise using VCA within the analysis of mobility.

Analysis of stability showed good or stronger agreement in general, but it was not significant in an exercise (cat and cow). An anteroposterior postural sway during the side spine stretch showed a good correlation of ICC > 0.668, where pelvis stability showed an excellent correlation of ICC > 0.763. VCA-based tele-assessment using only one web camera showed limitations in evaluating three-dimensional motions. Although it is possible to overcome some of the limitations if the evaluations are repeated with different views, evaluations of the other two planes remain limited. Difficulties in assessing stability exist compared to mobility evaluation, which only assesses the amount of motion, whereas stability evaluation requires assessing the ability to fix and limit unwanted movement.

The analysis of symmetry showed weak agreement in comparison to the other factors. The bridge and toe taps showed moderate correlation but showed nonsignificant results for the remaining exercises. Clinicians account for the differences among individuals and evaluate movement by comparing the symmetry between the two sides of the body. Different views of the camera are crucial for comparing the sides. Weak ICCs in the symmetry factor in this study are thought to be caused by a one-sided camera view. In this study, the exercise only displayed the sagittal plane of the body. During the side spine stretch, pelvic tilt from one side was observable, but the other side was not. Therefore, it appeared to be difficult to confirm body symmetry. Likewise, only one side of the quadruped exercise was observable, making it difficult to evaluate symmetry. Thus, observation of the movement in the frontal and transverse planes is needed. Although there may be a difference in quality of the assessment and supplements to be made on the factors of assessment, the results of this study have revealed feasibility of assessment and analysis during telerehabilitation as an alternative approach of direct contact assessment, which requires either patients to visit a hospital or therapists to visit the patients.

The inter-rater reliability of the tele-assessment displayed good agreement (ICC = 0.744) in the mean score of the eight raters. Mobility showed moderate correlations of ICC > 0.541 for the side spine stretch, >0.560 for bridge, >0.583 for toe taps, and >0.639 for quadruped leg raise and a good correlation in cats and cow with ICC > 0.801. Mobility evaluation showed strong accuracy and inter-rater consistency, thereby confirming its usability. The ICC of stability evaluation was significant, displaying moderate inter-rater reliability with an ICC > 0.634 for bridge, >0.711 for toe taps, >0.537 for quadruped leg raise, and >0.574 for cat and cow. The ICC of symmetry showed a moderate correlation, with ICC > 0.551 for bridge and >0.678 for quadruped leg raise.

A good level of ICC from evaluations preceded by experts with different experiences and fields with a limited view of movement from a webcam showed the feasibility of tele-assessment. However, the inter-rater reliability showed limitations owing to the lack of camera views, as it was also present in the validity results. Future studies should include guidelines to minimize the limitations of protocols and limitations of tele-assessment.

A previous study confirmed that the tele-assessment, including gait analysis, muscle strength tests, and joint range of motion, were accurate and reliable, thereby supporting the results of this study [3,25,26,27]. Tele-assessment was confirmed to be valid and reliable, with 68% exact agreement with non-articular lower limb musculoskeletal diagnoses and 80% exact agreement with musculoskeletal ankle diagnosis [4,28]. Musculoskeletal assessment, including range of motion, functional assessment, muscle strength, balance, and gait, showed overall good concurrent validity and excellent reliability [3,10,25,26,29]. The efficacy of tele-assessment has also been confirmed in clinical assessments of lower back pain and shoulder and elbow disorders [25,29,30,31]. Steele et al. [31] reported that telerehabilitation showed a strong satisfaction with both the therapist and patient, similar to the existing in-contact assessment. The COVID-19 pandemic has created a strong need for telehealthcare, and the results of this study would support the feasibility of telerehabilitation.

## 5. Conclusions

This study confirmed that it is a valid and reliable method of tele-assessment using video conferencing applications and showed feasibility as an alternative to the existing face-to-face examination.

## Figures and Tables

**Table 1:** List of exercise components analyzed with 3D motion analysis and tele-assessment using a video conferencing application.

Exercises	Components	3D Motion Analysis	Tele-Assessment Using Video Conferencing Application
Side spine stretch	Mobility (angle)	Angle of trunk motion	Was there a fine movement of the trunk?
	Trunk stability (angle)	Angle of forward and backward motion of trunk	Was there a forward and backward movement of the trunk?
	Pelvis stability (angle)	Angle of left-and-right motion of pelvis	Was there a left-and-right movement of the trunk?
	Symmetry (angle)	Angle difference of trunk motion between left and right	Was there a left-and-right symmetry in movement?
Bridge	Mobility (angle)	Angle of trunk extension	Did legs and trunk move in a line?
	Stability (mm)	Distance of leg sway	Were there any movements of the legs?
	Symmetry (angle)	Angle of left-and-right motion of pelvis	Was there a tilting of the pelvis to the sides?
Toe taps	Mobility (angle)	Angle of knee joint motion	Was there a fine movement of the hip joint while controlling knee joint movement? (reverse coded)
	Stability (angle)	Angle of the pelvis	Was there a sway of the pelvis?
	Symmetry (mm)	Distance between the knees	Was the distance between the knees constant?
Quadruped leg raise	Thigh mobility—starting position (angle)	Angle of thigh	Was the femur aligned vertically after coming back to the starting position?
	Thigh mobility—end position (angle)	Angle of thigh	Were the femur and lower leg parallel when they were lifted at the end position?
	Stability (mm)	Distance of femur sway	Was there any sway in the supporting leg?
	Symmetry (angle)	Angle difference of hip joints	Was there a left-and-right movement symmetry in the legs?
Cat and cow	Mobility (angle)	Angle of pelvis motion	Was there a fine movement of the pelvis?
	Hip joint stability (angle)	Angle of femur	Did the supporting leg maintain at 90°? (reverse coded)
	Leg stability (mm)	Distance of femur sway	Was there any sway in the supporting leg?

**Table 2:** Concurrent validity of the raters in tele-assessment using a video conference application.

Exercise	Components	3D Motion Analysis		Tele-Assessment		ICC (95% CI)	CV%	95% LOA
		Mean	SD	Mean	SD			
Side spine stretch	Mobility	13.95	5.97	6.39	0.90	0.884	33.80	0.757~0.945
	Trunk stability	5.15	1.60	4.32	0.75	0.668	24.82	0.303~0.842
	Pelvis stability	5.25	4.43	3.40	0.75	0.763	59.80	0.503~0.887
	Symmetry	1.02	0.50	6.61	0.52	0.396	13.28	−0.267~0.712
	Mobility	171.80	6.06	6.72	1.09	0.820	4.01	0.622~0.914
Bridge	Stability	156.97	58.14	3.92	1.12	0.753	36.83	0.481~0.882
	Symmetry	1.10	1.25	4.17	1.25	0.667	47.35	0.301~0.841
	Mobility	26.44	12.46	5.26	1.26	0.573	43.26	0.252~0.761
Toe taps	Stability	1.00	0.42	6.03	1.28	0.628	24.13	0.351~0.804
	Symmetry	249.67	68.60	5.16	0.98	0.564	27.30	0.262~0.768
	Thigh mobility—starting position	70.41	7.99	5.37	1.12	0.668	12.03	0.303~0.842
Quadruped leg raise	Thigh mobility—end position	7.16	5.62	5.07	0.97	0.544	53.86	0.234~0.753
	Stability	9874.67	1702.60	5.98	1.11	0.611	17.24	0.183~0.815
	Symmetry	2.67	1.78	5.28	1.10	−0.387	36.25	−1.915~0.339
	Mobility	42.09	10.57	5.83	1.40	0.811	24.98	0.603~0.910
Cat and cow	Hip joint stability	47.24	20.08	6.70	0.81	−0.045	38.73	−1.197~0.502
	Leg stability	466.03	185.16	5.48	0.73	0.193	39.42	−0.694~0.616

Values are expressed as mean ± standard deviation (SD). ICC, intraclass correlation coefficient; 95% CI, 95% confidence interval; CV%, coefficient of variation; 95% LOA, 95% limits of agreement.

**Table 3:** Inter-rater reliability of the raters in tele-assessment using video conference application.

Exercise	Components	R1	R2	R3	R4	R5	R6	R7	R8	ICC (95% CI)	95% LOA
Side spine stretch	Mobility (angle)	5.87	9.67	6.87	5.33	7.60	4.93	4.47	7.00	0.541	0.793~0.821
	Trunk stability (angle)	2.60	1.47	4.53	5.27	5.60	5.20	5.60	5.27	0.181	−0.302~0.622
	Pelvis stability (angle)	3.53	1.07	4.00	4.67	1.60	4.67	4.27	2.80	0.271	−0.116~0.651
	Symmetry (angle)	6.27	9.13	6.80	4.67	6.73	6.13	6.53	7.33	0.145	−0.292~0.554
Bridge	Mobility (angle)	7.00	9.73	7.80	3.93	7.93	5.20	5.47	7.47	0.560	0.234~0.807
	Stability (mm)	3.87	4.27	3.67	4.27	2.00	5.27	4.13	2.67	0.634	0.319~0.850
	Symmetry (angle)	4.20	2.60	5.73	4.53	2.60	4.73	4.80	2.93	0.551	0.179~0.814
	Mobility (angle)	5.80	9.20	3.87	3.27	5.47	5.20	4.00	6.80	0.583	0.261~0.820
Toe taps	Stability (angle)	6.60	8.53	4.53	4.33	6.73	5.60	5.87	7.33	0.711	0.437~0.882
	Symmetry (mm)	4.80	4.33	4.33	4.33	5.53	5.80	7.00	6.60	0.423	−0.013~0.754
	Thigh mobility—starting position (angle)	5.00	5.67	4.87	4.13	6.53	5.73	5.67	6.47	0.639	0.314~0.854
Quadruped leg raise	Thigh mobility—end position (angle)	5.53	4.40	5.13	4.40	5.80	4.60	5.60	6.27	0.478	0.009~0.789
	Stability (mm)	6.80	6.00	6.00	6.80	6.73	5.60	3.93	6.07	0.537	0.143~0.810
	Symmetry (angle)	6.13	4.60	4.60	4.20	5.40	5.73	6.27	6.20	0.678	0.388~0.870
	Mobility (angle)	6.27	8.00	4.67	4.47	7.67	5.33	4.40	4.87	0.801	0.575~0.923
Cat and cow	Hip joint stability (angle)	7.20	8.47	5.73	5.13	7.80	6.40	6.13	6.53	0.574	0.249~0.817
	Leg stability (mm)	6.13	8.20	4.93	4.53	4.33	6.00	4.20	4.73	0.242	−0.135~0.628
Total										0.744	0.694~0.789

Values are expressed as the mean. R, raters; ICC, intraclass correlation coefficient; 95% CI, 95% confidence interval; 95% LOA, 95% limits of agreement.

## Data Availability

Not applicable.
